# Social, Clinical and Microbiological Differential Characteristics of Tuberculosis among Immigrants in Spain

**DOI:** 10.1371/journal.pone.0016272

**Published:** 2011-01-20

**Authors:** José-María García-García, Rafael Blanquer, Teresa Rodrigo, Joan A. Caylà, José A. Caminero, Rafael Vidal, Martí Casals, Juan Ruiz-Manzano, the Working Group on Completion of Tuberculosis Treatment in Spain

**Affiliations:** 1 Programa Integrado de Investigación en Tuberculosis (PII TB) de la Sociedad Española de Neumología y Cirugía Torácica (SEPAR), Barcelona, Spain; 2 Hospital San Agustín, Avilés, Spain; 3 Hospital Universitario Dr Peset de Valencia, Valencia, Spain; 4 Fundación Respira de la SEPAR, Barcelona, Spain; 5 Unidad de Investigación de Tuberculosis de Barcelona, Servicio de Epidemiología de la Agencia de Salud Pública de Barcelona, Barcelona, Spain; 6 Hospital General Universitario de Gran Canaria Dr Negrín, Canary Islands, Spain; 7 International Union Against Tuberculosis and Lung Disease, Paris, France; 8 CIBER de Enfermedades Respiratorias (CIBERES), Barcelona, Spain; 9 Hospital Vall D'Ebrón de Barcelona, Barcelona, Spain; 10 CIBER de Epidemiología y Salud Pública (CIBERESP), Barcelona, Spain; 11 Departament de Salut Pública, Universitat de Barcelona, Barcelona, Spain; 12 Departament de Ciencies Basiques, Universitat Internacional de Catalunya, Barcelona, Spain; 13 Hospital Universitario Germans Trías y Pujol de Badalona, Badalona, Spain; Fundació Institut Germans Trias i Pujol; Universitat Autònoma de Barcelona CibeRES, Spain

## Abstract

**Background:**

To identify the differential tuberculosis (TB) characteristics within the immigrant population with respect to natives in Spain.

**Methodology/Principal Findings:**

A prospective cohort study design was implemented to examine the TB cases diagnosed and starting standard antituberculous treatment in Spain, between January 1st 2006 and March 31st 2007. A logistic regression analysis was performed to determine differential characteristics. 1,490 patients were included in the study population, 1,048 natives and 442 (29.7%) immigrants. According to the multivariate analysis, the following variables were significantly associated with immigrant TB cases: younger age (OR = 3.79; CI:2.16–6.62), living in group situation (OR = 7.61; CI:3.38–12.12), lower frequency of disabled (OR:0.08; CI:0.02–0.26) and retired (OR:0.21; CI:0.09–0.48) employment status, lower frequency of pulmonary disease presentation (OR = 0.47; CI:0.24–0.92), primary or emergency care admission (OR = 1.80; CI:1.05–3.06 and OR = 2.16; CI:1.36–3.45), drug resistance (OR = 1.86; CI:1.01–3.46), treatment default (OR:2.12; CI:1.18–3.81), lower frequency of alcohol and cigarette consumption (OR = 2.10; CI:1.42–3.11 and OR = 2.85; CI:2.10–3.87 respectively), more directly observed treatment (OR = 1.68; CI:1.04–2.69), and poor understanding of TB disease and its treatment (OR = 3.11; CI:1.86–5.20). The low percentage of primary MDR-TB in the native population (0.1% vs. 2.2% of immigrants) should be noted.

**Conclusions/Significance:**

The differences show the need to introduce specific strategies in the management of TB within the immigrant population, including the improvement of social and work conditions.

## Introduction

The World Health Organization (WHO) estimated a tuberculosis (TB) annual incidence of 9.4 million cases (139/100,000 inhabitants) in 2008, of which 57% of pulmonary cases were smear-positive, 15% were co-infected with HIV and 11% were cases of multi-drug resistant TB (MDR-TB), defined as resistance to at least isoniazid and rifampin [1].

The global TB prevalence has progressively decreased in high-income countries, however HIV co-infection, immigration from high TB burden countries for economic reasons, MDR-TB and overcrowding within poor communities of large cities have hindered further decline. The decrease in TB rates began to taper in many industrialized countries and TB rates in some even started to increase in the early 1990s. However, incidence among the native population continued to decrease, resulting in a higher proportion of foreign-born cases. This trend has been documented in European countries such as Denmark, Holland, Sweden, the United Kingdom and Switzerland, where foreign-born cases comprise more than half of TB cases, with incidences such as 100/100,000 among immigrants versus 15/100,000 among the native population [2].

MDR-TB is a growing problem worldwide [3]. In 2008, an estimated 390.000 to 510,000 cases of MDR-TB emerged globally. Among all global incident TB cases, 3.6% are believed to have MDR-TB. Almost 50% of MDR-TB cases worldwide are estimated to occur in China and India, and another 7% in Russia and the former Soviet countries [4]. Immigrants in Europe may be from these and other high MDR-TB burden countries. The prevalence of MDR-TB among new TB cases ranged from 0% in some countries to 22.3% [3], [5]. In 2008, MDR-TB caused an estimated 150,000 deaths [4]. The global expenditure in the diagnosis and treatment of MDR-TB and extensively drug-resistant TB (XDR-TB), defined as MDR-TB plus resistance to fluroquinolones and at least one second-line injectable drug, were estimated at 700 million US dollars (USD) for 2009 and the cost of treatment for a MDR-TB case is estimated at 10,000USD versus 100USD for a drug-susceptible TB case [6].

A study performed in Spain by the Integrated Investigation Programme in TB (PII TB) of the Spanish Respiratory and Thoracic Surgery Society (SEPAR) showed a considerable increase in foreign-born TB cases, reaching 30% of the total cases [7]. In Barcelona, a Spanish city which has experienced a constant influx of immigrants, the percentage of foreign-born cases increased from 5% to 50% between 1995 and 2008 [8].

The objective of this study was to determine the differential social, clinical and microbiological characteristics between native and immigrant cases for the implementation of stricter control measures during treatment follow-up to monitor treatment completion among this group of patients.

## Methods

### Participants and description of procedures

This multi-center prospective cohort study of TB cases was supported by 61 collaborators, mainly respiratory and infectious disease physicians, from 53 health centers in the different regions of Spain. The study population included a cohort of TB patients of 18 years of age or older diagnosed between January 1^st^ 2006 and March 31^st^ 2007, who started standard anti-TB treatment with rifampin and isoniazid during 6 months, and pyrazinamide with ethambutol (defined as four drugs) or without ethambutol (defined as three drugs) for the first two months. Patients with known previous drug resistance or those for whom standard anti-TB treatment was contraindicated were excluded. Study data included socio-demographic information (patients born outside of Spain were assumed to have moved mainly for economic reasons), smoking (including smokers of one or more cigarettes per day and ex-smokers), drug and alcohol habits (men consuming over 280 g of alcohol per week, and women over 168 g, were considered alcoholics). Users of intravenous heroin and/or cocaine were classified as intravenous drug users (IVDU), anthropometrics, clinical history, diagnostic methods, drug susceptibility testing and results, anti-TB treatment, clinical response, and treatment adherence and outcome. This information was collected using electronic case reports which were available to collaborating investigators on the SEPAR website. All cases were followed according to an evaluation schedule (Table 1).

**Table 1:** Patient evaluation calendar.

	Visit 1Diagnosis	Visit 22 Months	Visit 36 Months	Visit 4*9,12,18 Months(Optional)
**Inclusion/Exclusion criteria**	X			
**Socio-demographic data**	X			
**Smoking/alcohol habits**	X			
**Anthropometrics**	X	X	X	X
**Clinical history**	X			
**Diagnostic methods**	X			
**Drug treatment**	X	X	X	X
**Clinical response**		X	X	X
**Treatment adherence**		X	X	X
**Sputum sample collection**	X	X	X	X
**Drug susceptibility testing**	X			
**Treatment outcome**			X	X

*If treatment is continued for more than 6 months.

### Ethics Statement

In accordance with the International Directory for Ethical Revision of Epidemiological Studies (Council for the International Organizations of Medical Sciences - CIOMS, Geneva, 1991) and the Spanish Epidemiology Society recommendations on ethical aspects of epidemiological research, this study was submitted to the Teknon Medical Center Investigation Ethical Committee in Barcelona for evaluation. Verbal informed consent was obtained for patient participation. All registries with patient identification information were handled in a confidential manner and in accordance with the Spanish Law 15/1999 on the Protection of Personal Character Data.

### Statistical methods

A descriptive study was carried out on qualitative and quantitative variables to characterize the study population. Quantitative data are shown as medians and interquartile ranges and qualitative data as a percentage. Proportions were compared between groups using ×2 tests, and when pertinent, the two-sided Fisher test. The Mann-Whitney U test was used to compare the values of diagnostic delay. Association was determined by *odds ratio* (OR) and their 95% confidence intervals (CI). The association of TB characteristics with the native or immigrant populations was analyzed by a stepwise logistic regression including the factors associated on a bivariate level. The test of Hosmer and Lemeshow was used to check the goodness-of-fit of the model. A p-value of <0.05 was considered significant. All of the analyses were completed using SPSS Statistical Package, version 13.0 (SPSS Inc, Chicago, IL, USA).

## Results

Of the 1,500 patients enrolled during the study period, 10 were excluded because they did not satisfy inclusion criteria and the final total of analyzed cases was 1,490. Four hundred forty-two (29.7%) were immigrants from 48 countries; the largest proportions were from Romania (13.9%), Bolivia (13.5%), Morocco (11.5%), Pakistan (9.5%), Ecuador (7.5%), Senegal (4.6%), Colombia (4.4%), and Peru (4.4%).

In a bivariate analysis, the following TB characteristics were significantly associated with immigrant TB cases: lower frequency of disabled and retired employment status, pulmonary disease presentation, alcohol consumption, smokers. However the following characteristics were higher among immigrants: aged between 18–30 or 31–50 years, unemployment, living in group situation, primary care or emergency care admission (instead of specialized care), treatment with 4 drugs, drug resistance, treatment default, directly observed treatment (DOT) implementation, and poor understanding of TB disease and its treatment according to the perception of the doctor in charge of the patient. There were no statistically significant differences in sex, previous TB treatment, injection drug use (IDU), diagnostic tests performed, HIV infection, or diagnostic delay (Table 2).

**Table 2:** Tuberculosis characteristics in native and immigrants of Spain.

VARIABLES		NativeN (%)	ImmigrantN (%)	p value	OR	95% CI
**SEX**	MaleFemale	663 (64.7)362 (35.3)	257(60.2)170 (39.8)	0.105	0.821	0.65 – 1.04
**AGE (YEARS)**	18–3031–50>50	285 (27.2)395 (37.7)368 (35.1)	212 (48.0)201 (45.5)29 (6.6)	**<0.001** **<0.001**	**9.43** **6.45**1	**6.21 – 14.33**4.26 – 9.7
**EMPLOYMENT**	EmployedDisabled^1^UnemployedRetiredUnknown	591(56.4)64 (6.1)152 (14.5)210 (20.0)31 (3.0)	310 (70.1)4 (0.9)105 (23.8)10 (2.3)13 (2.9)	**<0.001** **0.058** **<0.001**0.508	1**0.11** **1.31** **0.09**0.79	**0.04 – 0.33** **0.99 – 1.75** **0.04 – 0.17**0.41 – 1.55
**LIVING SITUATION**	AloneHomeless or incarceratedGroup^2^FamilyUnknown	128 (12.2)33 (3.1)36 (3.4)835 (79.7)16 (1.5)	29 (6.7)21 (4.8)153 (34.6)227 (51.4)12 (2.7)	**0.003** **<0.001** **0.003**0.728	**0.35** **1** **6.67** **0.42**1.17	**0.18 – 0.70** **3.46 – 12.87** **0.24 – 0.75**0.46 – 2.97
**CLINICAL CENTER**	Primary careEmergency careUnknownSpecialist	185 (17.7)445 (42.5)245 (23.4)173 (16.5)	83 (18.8)237 (53.6)85 (19.2)37 (8.4)	**0.001** **<0.001** **0.028**	**2.09** **2.49** **1.62**1	**1.35 – 3.25** **1.68 – 3.67** **1.05 – 2.49**
**PREVIOUS TREATMENT**	NoYesUnknown	929 (88.6)92 (8.8)27 (2.6)	391 (88.5)39 (8.8)12 (2.7)	0.9710.905	0.9911.04	0.67 – 1.470.48 – 2.27
**IDU** 3	NoUnknownYes	616 (58.8)416 (39.7)16 (1.5)	256 (57.9)181 (41.0)5 (1.1)	0.5820.524	1.331.391	0.48 – 3.660.502 – 3.85
**DIAGNOSTIC METHODS**	Smear (+)Smear (−)/Culture. (+)Smear (−)/Culture (−)Others	611 (58.3)258 (24.6)128 (12.2)51 (4.9)	265 (60.0)76 (17.2)76(17.2)25 (5.7)	0.6310.0660.500	0.8850.6011.211	0.53–1.450.34–1.030.69–2.11
**SITE OF TUBERCULOSIS**	Extra-pulmonaryMixedPulmonaryUnknown	99 (9.4)40 (3.8)899 (85.8)10 (1.0)	60 (13.6)27 (6.1)350 (79.2)5 (1.1)	0.718**0.032**0.618	0.891**0.57**0.74	0.50 – 1.61**0.34 – 0.95**0.22 – 2.40
**TREATMENT**	3 Drugs4 DrugsUnknown	698 (66.6)316 (30.2)34 (3.2)	72 (16.3)333 (75.3)37 (8.4)	**<0.001** **<0.001**	1**10.21** **10.55**	**7.66 – 13.61** **6.24 – 17.83**
**DRUG SUSCEPTIBILITY**	SusceptibleResistant^4^Unknown	801(76.4)40 (3.8)207 (19.8)	318 (71.9)31 (7.0)93 (21.0)	**0.007**0.382	1**1.95**1.13	**1.20 – 3.17**0.85 – 1.49
**TREATMENT DEFAULT**	NoYesUnknown	963 (91.9)41 (3.9)44 (4.2)	369 (83.5)51 (11.5)22 (5.0)	**<0.001**0.321	1**3.24**1.30	**2.11 –4.98**0.77 – 2.20
**ALCOHOL CONSUMPTION**	NoYesUnknown	694 (66.2)317 (30.2)37 (3.5)	370 (83.7)58 (13.1)14 (3.2)	**<0.001** **0.035**	**2.91** **1** **2.06**	**2.14 – 3.96** **1.05 – 4.06**
**SMOKING** 5	NoYesUnknown	406 (38.7)630 (60.1)12 (1.1)	270 (61.1)167 (37.8)5 (1.1)	**<0.001**0.402	**2.50** **1**1.57	**1.99 – 3.15**0.54 – 4.52
**HIV INFECTION**	NoYesUnknown	717 (68.4)47 (4.5)284 (27.1)	343 (77.6)19 (4.3)80 (18.1)	0.5470.228	1.1810.69	0.68 –2.040.38 – 1.25
**DOT** 6	NoYes	956 (91.2)92 (8.8)	382 (86.4)60 (13.6)	**<0.001**	1**1.63**	**1.15 –2.30**
**COMPRE-** **HENSION** 7	WellDifficultUnknown	928 (88.5)74 (7.1)46 (4.4)	338 (76.5)65 (14.7)39 (8.8)	**<0.001** **<0.001**	**1** **2.41** **2.32**	**1.69 – 3.44** **1.49 – 3.63**
**DIAGNOSTIC DELAY** 8	Days; median (IQR)	48 (24–92)	42 (21–91)	0,074		

1 Disabled: lacking one or more of the physical or mental abilities that most people have.

2 Group living or living in a group: People of different families who live together in the same flat.

3 IDU: Intravenous Drug Use.

4 Resistant at least to one drug.

5 In relation to tobacco: no (never smokers), yes (current or ex-smokers).

6 DOT: Directly observed treatment.

7 Comprehension or understanding of TB: understanding of disease and its treatment according the perception of the doctor in charge of the patient.

8 Diagnostic Delay: Median number of days between symptom onset and treatment initiation.

Cohort of 1,490 patients. Bivariate analysis.

According to the multivariate analysis, the following variables were significantly associated with immigrant TB cases compared to native cases: lower frequency of disabled and retired employment status, alcohol and cigarette consumption, pulmonary disease presentation, and higher frequency of age between 18–30 or 31–50 years, a group living situation, primary or emergency care admission, drug resistance, treatment default, DOT and poor understanding of TB disease and its treatment (Table 3). The highest odds ratios were calculated for living in group situation, aged between 18–30 years or 31–50 years and for poor understanding of TB disease and its treatment. A higher rate of successful treatment outcome using standard treatment was found within the native population than among immigrants (91.9% of natives and 83.5% of immigrants) (Table 4). Similarly, treatment default was higher within the immigrant population.

**Table 3:** Tuberculosis characteristics in native and immigrants of Spain.

VARIABLES		NativeN (%)	ImmigrantN (%)	p value	OR	95% CI
**AGE (YEARS)**	18–3031–50>50	285 **(27.2)**395 **(37.7)**368 (35.1)	212 **(48.0)**201 **(45.5)**29 (6.6)	**<0.001** **<0.001**	**3.79** **3.53**1	**2.16–6.62** **2.03–6.14**
**EMPLOYMENT**	EmployedDisabledUnemployedRetiredUnknown	591(56.4)64 **(6.1)**152 (14.5)210 **(20.0)**31 (3.0)	310 (70.1)4 **(0.9)**105 (23.8)10 **(2.3)**13 (2.9)	**<0.001**0.683**<0.001**0.358	1**0.08**1.07**0.21**0.69	**0.02–0.26**0.74–1.55**0.09–0.48**0.32–1.50
**LIVING SITUATION**	AloneHomeless or incarceratedGroupFamilyUnknown	128 (12.2)33 (3.1)36 **(3.4)**835 (79.7)16 (1.5)	29 (6.7)21 (4.8)153 **(34.6)**227 (51.4)12 (2.7)	0.324**<0.001**0.2230.139	0.651**7.61**0.632.42	0.28–1.51**3.38–12.12**0.30–1.320.75–7.81
**CLINICAL CENTER**	Primary careEmergency careUnknownSpecialist	185 **(17.7)**445 **(42.5)**245 (23.4)173 (16.5)	83 **(18.8)**237 **(53.6)**85 (19.2)37 (8.4)	**0.030** **0.001**0.126	**1.80** **2.16**1.501	**1.05–3.06** **1.36–3.45**0.89–2.54
**SITE OF TUBERCULOSIS**	Extra-pulmonaryMixedPulmonaryUnknown	99 (9.4)40 (3.8)899 **(85.8)**10 (1.0)	60 (13.6)27 (6.1)350 **(79.2)**5 (1.1)	0.700**0.027**0.108	0.861**0.47**0.26	0.39–1.85**0.24–0.92**0.05–1.33
**DRUG SUSCEPTIBILITY**	SusceptibleResistantUnknown	801(76.4)40 **(3.8)**207 (19.8)	318 (71.9)31 **(7.0)**93 (21.0)	**0.047**0.584	1**1.86**1.10	**1.01–3.46**0.77–1.56
**TREATMENT DEFAULT**	NoYesUnknown	963 (91.9)41 **(3.9)**44 (4.2)	369 (83.5)51 **(11.5)**22 (5.0)	**0.012**0.376	1**2.12**1.40	**1.18–3.81**0.66–2.97
**ALCOHOL CONSUMPTION**	NoYesUnknown	694 **(66.2)**317 (30.2)37 (3.5)	370 **(83.7)**58 (13.1)14 (3.2)	**<0.001**0.070	**2.10**12.20	**1.42–3.11**0.93–5.18
**SMOKING**	NoYes^1^Unknown	406 **(38.7)**630 (60.1)12 (1.1)	270 **(61.1)**167 (37.8)5 (1.1)	**<0.001**0.370	**2.85**11.79	**2.10–3.87**0.49–6.47
**DOT**	NoYes	956 (91.2)92 **(8.8)**	382 (86.4)60 **(13.6)**	**0.031**	1**1.68**	**1.04–2.69**
**COMPRE-** **HENSION**	WellDifficultUnknown	928 (88.5)74 **(7.1)**46 (4.4)	338 (76.5)65 **(14.7)**39 (8.8)	**<0.001**0.111	1**3.11**1.70	**1.86–5.20**0.88–3.28

1 Including ex smokers.

Cohort of 1,490 patients. Multivariate analysis.

**Table 4:** Treatment outcome distribution among native and immigrant tuberculosis cases*.

TREATMENT OUTCOME	N NATIVE (%)	N IMMIGRANT (%)	N TOTAL (%)
**Cured**	583 (**55.6**)	209 (**47.3**)	792 (53.2)
**Completed treatment**	380 (**36.3**)	160 (**36.2**)	540 (36.2)
**Incomplete**	1 (**0.1**)	1 (**0.2**)	2 (0.1)
**Transfer out**	16 (**1.5**)	17 (**3.8**)	33 (2.2)
**Default**	13 (**1.2**)	14 (**3.2**)	27 (1.8)
**Died**	23 (**2.3**)	4 (**0.9**)	27 (1.8)
**Lost to follow-up**	28 (**2.7**)	37 (**8.4**)	65 (4.4)
**Other**	4 (**0.4**)	0 (**0.0**)	4 (0.3)
**TOTAL**	**1048 (100.0)**	**442 (100.0)**	**1490 (100.0)**

*Successful treatment outcome is the sum of cured cases plus case of completed treatment.

Drug susceptibility was analyzed in 1,046 patients (70.2%), of whom 745 were natives and 301 were immigrants. Of the 959 patients who had never previously received TB treatment, 682 were natives and 277 were immigrants. The low percentage of primary MDR-TB in the native population (0.1%vs 2.2% of immigrants) and the absence of mono-resistance to rifampin in both groups of patients should be noted. Differences between global percentages of drug resistance (18.3% of immigrants vs. 7.9% of native cases) and primary drug resistance (16.6% vs. 6.6%, respectively) were statistically significant (p<0.001 for both), as well as for rates of MDR TB (Table 5). Drug susceptibility testing to fluoroquinolones and second-line injectable drugs was not performed routinely; so it was performed in few patients of our study population. Six patients presented criteria for XDR-TB, 2 among native cases and 4 among immigrant cases.

**Table 5:** Distribution of drug resistance among native and immigrant tuberculosis patients with drug susceptibility testing performed.

RESISTANCE		N NATIVEN/total (%)	N IMMIGRANT N/total (%)	p value	OR	95% CI
**Global**	**Total** **Isoniazid** **Rifampin**PyrazinamideEthambutolStreptomycin**MDR-TB**	59/745 **(7.9)**27/745 **(3.6)**2/745 **(0.3)**6/745 (0.8)5/745 (0.7)19/745 (2.6)2/745 **(0.3)**	55/301 **(18.3)**26/301 **(8.6)**8/301 **(2.7)**4/301 (1.3)3/301 (1.0)14/301 (4.7)8/301 **(2.7%)**	**<0.001** **<0.001** **0.001**0.8170.8710.162**0.001**	**2.60** **2.5** **10.14** **10.14**	**1.72–3.93** **1.39–4.54** **1.99–69.50** **1.99–69.50**
**Primary**	**Total** **Isoniazid** **Rifampin**PyrazinamideEthambutolStreptomycin**MDR-TB**	45/682 **(6.6)**22/682 **(3.2)**1/682 **(0.1)**5/682 (0.7)4/682 (0.6)13/682 (1.9)1/682 **(0.1)**	46/277 **(16.6)**23/277 **(8.3**)6/277 **(2.2)**2/277 (0.7)3/277 (1.1)12/277 (4.3)6/277 **(2.2)**	**<0.001** **<0.001** **0.002**0.990.6890.05**0.002**	**2.82** **2.72** **15.08** **15.08**	**1.7 8–4.47** **1.43–5.16** **1.80–33.87** **1.80–33.87**
**Acquired**	TotalIsoniazidRifampinPyrazinamideEthambutolStreptomycin**MDR-TB**	14/63 (22.2)5/63 (7.9)1/63 (1.6)1/63 (1.6)1/63 (1.5)6/63 (9.5)1/63 **(1.6)**	9/24 (37.5)3/24 (12.5)2/24(8.3)2/24 (8.3)0/24(0.0)2/24 (8.3)2/24 (**8.3**)	0.240.800.370.37110.37		

MDR: Drug resistance to at least isoniazid (H) and rifampin (R).

## Discussion

This multi-center study of TB diagnosed patients who started standard anti-TB treatment resulted in a considerable percentage of the foreign-born cases (29.7%) and demonstrated that particular characteristic differences, personal, social as well as concerning treatment follow-up and drug resistance, exist when compared to native TB cases.

With respect to TB characteristics among the immigrant and native populations, two recent publications did not find significant differences of TB clinical presentation or risk factors, including HIV co-infection [9], [10]. The studies also showed that undocumented immigrants have a larger diagnostic delay and present more severe symptoms and advanced disease, even when no statistically significant differences existed between the native and documented immigrant populations. According to the authors, all recently arriving immigrants should been screened, despite absence of symptoms [9], [10]. Study results from Spain are somewhat heterogeneous and most discuss immigrant TB case characteristics only. Some mention higher prevalence of HIV co-infection [11], [12] or higher latent TB infection rates [13], while others vary in the predominant clinical forms of TB [11], [12], [14], rates of drug resistance [13], or the proportion of lost to follow-up cases [15].

A significant difference in age existed in our study between immigrant cases, who were mainly under 50 years old, and native cases, who were predominantly older. These results are consistent with local and international publications [11], [16], [17] and parallel the demographic characteristics of the total immigrant population. Given the recent influx of immigrants to Spain and the young age of those who migrate most of them for economical reasons, this age difference between immigrant and native TB cases is logical. A group living situation was also more frequent among immigrants due to poor economical circumstances and is also a documented risk factor for TB transmission [18]. We also found that immigrant TB cases were admitted to emergency care more than native cases; although the health system in Spain is universal this type of service is more accessible than a specialist clinic (where an appointment is necessary) to persons with frequent address changes or an undocumented legal status, both common characteristics in the foreign-born population. A similar result was observed in a study carried out in Madrid [11]. Immigrant TB cases also had lower average cigarette and alcohol consumption than their native counterparts, as shown in Switzerland as well [9]. We think that social and religious customs, as well as poor economical status within the immigrant population, could have contributed to their low consumption. Immigrant TB cases also had more difficulty understanding TB disease and treatment, most likely for language, social, and economical reasons. TB Programs should be adapted to migration changes [18]. The use of community health workers, who play the role of translators and culture mediators, has been demonstrated as useful to minimize this problem [19].

Regarding treatment follow-up and final outcome, the native population has a higher proportion of successful treatment completion (cured patients plus those who complete treatment) than the foreign-born population, even though successful treatment completion was high in both groups (91.9% of natives and 83.5% of immigrants but in this group is lower than the recommendation of more than 85%). Treatment default was also more frequent among the immigrant population, though it was not considered high (8.4%). Given these findings [13], [20], control measures, such as the use of DOT [13], [21], more exhaustive clinical follow-up with more visits, better communication between healthcare professionals in different regions to locate transfer-out and lost to follow-up patients, and continued efforts to integrate and assimilate this population should be reinforced.

Drug susceptibility data was available in 70.2% of the study population because only some collaborating centers could systematically perform drug susceptibility testing for all new patients. However, the 1,046 patients with drug susceptibility testing results available for first-line drugs available is large sample, especially those 959 (682 natives and 277 immigrants) never receiving anti-TB drugs in the past (defined as primary drug resistance in this study). The inclusion criteria (only patients receiving a standard six months regimen) could have introduced a bias of the acquired resistance, because most of the patients who had previously received anti-TB treatment were excluded. The motive for this criteria was to determine the frequency of the use of three or four drugs for standard treatment and the characteristics of the patients taking these regimens. This bias is not a factor for primary resistance because all new cases were included in the study. Our results also reveal low primary MDR-TB resistance (0.1%) in natives, as shown with other previous studies published in Spain [13], [22], [23]. The primary MDR-TB rate is one of the lowest of the world [4]–[6]. Free healthcare assistance offered by the Spanish Health System and the widespread use of fixed-dose combinations for more than 30 years could have contributed to the low rate of drug resistance in Spain.

The percentage of any drug resistance (primary or acquired), including MDR-TB, was higher among immigrants. This difference was statistically significant for all the groups (global and primary, total, for rifampin, isoniazid and MDR-TB) except for the acquired resistance, most likely because of the previously mentioned bias. Differing patterns of primary resistance and that of isoniazid have also been documented in other studies [13], [16]. We also found a significant difference between the two populations regarding MDR-TB prevalence, despite the low number of cases in our population, as documented in other studies [16], [17]. Initial treatment using a four-drug regimen was most likely prescribed for immigrant cases because of a consensus recommendation [21] and our results support its continuation. Nonetheless, the difference in treatment prescription between immigrant and native cases will probably decrease because Spanish [24] and international TB treatment guidelines [25] now recommend that all cases should be initially treated with four drugs. The implementation of a prospective study examining primary and acquired drug resistance patterns is also necessary.

This study also provides two other interesting findings. The first is the absence of mono-resistance to rifampin in Spain, where all the strains with rifampin resistance were also resistant to at least isoniazid (MDR-TB). This strongly supports the use of rifampin resistance as a marker of MDR-TB [5]. Secondly, this study shows that XDR-TB cases do exist in Spain among both immigrant and native populations, despite the very low MDR-TB rate. Thus, drug susceptibility testing to fluoroquinolones and second-line injectables drugs should be performed systematically to all MDR-TB patients.

In this study we can also deduce that social determinants of health influences the epidemiological situation of TB among immigrants. In order to achieve a situation of equity, in these populations in Spain and in other countries with a high number of immigrants due mainly to economic reasons, several public health interventions are needed (favour their access to health system, directly observed therapy in some cases, TB programs with community health workers, etc) [21].

In conclusion, we have found that a considerable proportion of TB cases in Spain are immigrants and that significant differences exist between immigrant and native cases. We therefore reiterate that work and social conditions must be improved for the foreign community, as well as the availability of social workers and community health workers who address comprehension issues. Similarly, more exhaustive treatment follow-up efforts must be made, including the use of DOT, additional clinical visits, and more communication between healthcare professionals. Finally, standard TB treatment should start with a four-drug regimen and primary drug susceptibility testing should be preformed systematically to optimize treatment, stop further transmission, and above- all, prevent the emergence of drug resistances.
